# Clove Flower Extract (*Syzygium aromaticum*) Has Anticancer Potential Effect Analyzed by Molecular Docking and *Brine Shrimp Lethality Test* (BSLT)

**DOI:** 10.1155/2022/5113742

**Published:** 2022-09-05

**Authors:** Eduardus Bimo Aksono, Aprilia Cahya Latifah, Lucia Tri Suwanti, Kautsar Ul Haq, Herinda Pertiwi

**Affiliations:** ^1^Faculty of Veterinary Medicine, Airlangga University, Surabaya, Indonesia; ^2^Engineering and Life Sciences Institute, Airlangga University, Surabaya, Indonesia; ^3^Faculty of Science and Technology, Airlangga University, Surabaya, Indonesia; ^4^Faculty of Vocational Studies, Airlangga University, Surabaya 60115, Jawa Timur, Indonesia

## Abstract

Various anticancer medications have been discovered due to advances in the health care industry, but they have undesirable side effects. On the other hand, anticancer drugs derived from natural sources have low side effects, making them excellent for cancer therapy. This study aims to evaluate the effect of clove flower extract (*Syzygium aromaticum*) as a potential anticancer agent by determining grid-score values using molecular docking and LC50 values using the brine shrimp lethality test (BSLT) technique. As animal models, three hundred larvae of *Artemia salina* leach were divided into six groups. Each group has ten larvae that have undergone five replications. The clove flower extract concentration in the treatment media was 50 ppm (T1), 250 ppm (T2), 500 ppm (T3), 750 ppm (T4), 1000 ppm (T5), and 0 ppm (seawater) as the control. The probit analysis of *Artemia Salina* leach mortality percentage data. The results indicated that the clove flower extract (*Syzygium aromaticum*) is harmful to larvae with LC50 values of 227,1 g/ml or in the equation *y* = 2,8636 x – 1,7466 with an R2 value of 0.9062. According to molecular docking, eugenol acetate (grid-score −42.120834) has a close relationship with the cognate enzyme nitric oxide synthase (3E7G) based on its proximity to the grid score value (grid-score −61.271812). Therefore, clove flower extract has the potential to act as an anticancer medication. Based on the grid-score proximity, eugenol acetate is close to the homologous enzyme nitric oxide synthase (3E7G). Inhibition of nitric oxide synthase also shows a reduction in cancer cell proliferation.

## 1. Introduction

Some or all components of medicinal plants contain bioactive compounds that can be used to treat specific diseases. Leaves, fruit, flowers, seeds, rhizomes, stems, bark, and sap are all functional plant parts [1]. Cloves (*Syzygium aromaticum*) are an ancient spice plant known and utilized for centuries. The tree is native to the Maluku islands (Ternate and Tidore), Indonesia, which explorers formerly referred to as the spice islands [2, 3].

The essential oil content of clove flowers (10–20%), stems (5–10%), and leaves (1–4%) is substantial [4]. Additionally, clove essential oil has the highest quality due to its high yield and 80–90% eugenol content. Cloves are, therefore, multibeneficial and effective as food and beverage additives with high nutritional value, anticancer, antibacterial, antifungal, anti-inflammatory, antiproliferative, antifibrogenic, anti-insect, and analgesic properties [5]. In addition, cloves have a significant antioxidant activity due to their high eugenol concentration [6].

Cloves contain sesquiterpenes, monoterpenes, hydrocarbons, and phenolic substances as phytochemicals. The two most essential phytochemicals in clove oil are eugenol and caryophyllene. Eugenol has demonstrated anticancer properties against colon, stomach, breast, prostate, melanoma, and leukemia cancers, while caryophyllene has anticancer effects on pancreatic, cutaneous, lymphatic, and cervical malignancies [7].

The major component of clove oil, eugenol, is a possible contender for future development as an aid to current chemotherapeutic cancer therapies. Eugenol inhibits the growth and development of tumors, increases reactive oxygen species (ROS), induces apoptosis, and has genotoxic effects on many cancer cells [7]. Even Zari et al. [8] suggested that the anticancer action of eugenol was achieved via multiple mechanisms, including induced apoptosis, cell cycle arrest, suppression of proliferation, migration, angiogenesis, and metastasis of multiple cancer cell lines. Additionally, eugenol can be used as adjuvant therapy for individuals undergoing standard chemotherapy. This combination increases efficacy while decreasing toxicity. Several research findings indicate that eugenol plant extracts possess various biological properties, including antifungal, anticancer, and anti-inflammatory properties. For instance, as an anticancer agent, eugenol promotes apoptosis by downregulating E2F1/survivin in breast cancer [9].

Eugenol's chemical formula is C_10_H_12_O_2_, and its IUPAC name is 4-allyl-2- methoxyphenol. In addition, eugenol is also known as 4-alylguaikol, 1-allyl-4-hydroxy-3-methoxybenzene, cryophilic acid, 4-hydroxy-3-methoxyalyl benzene, and 2-methoxy-4- alkylphenol [8]. The percentage of eugenol in clove oil ranges from 70% to 96% [10], and its functional groups include allyl (-CH2-CH=CH2), methoxy (-OCH3), and phenol (OH) [11].

Various anticancer medications have been discovered due to advancements in the medical field. The primary objective of anticancer medications is to harm cancer cells without affecting normal cells selectively. This objective is typically attained, and very few anticancer medications now target specific cancer types [12, 13]. Recent studies have shown that plants containing phytochemical substances with anticancer capabilities are strongly connected with a decreased cancer risk. Moreover, natural products typically have few side effects, making them attractive candidates for cancer therapy [14]. However, there are few investigations into cloves as an anticancer agent.

This study is designed to examine the anticancer efficacy of cloves using the brine shrimp lethality test (BSLT). The brine shrimp lethality test is a straightforward benchtop bioassay that has provided favorable results for screening plant extracts for biological activity [15]. The brine shrimp is toxic to various chemicals and natural products; the toxicity test is based on the brine shrimp's death after exposure to varying plant extracts. This premise has been used in screening therapeutic plant extracts in the BSLT, as toxicology can be described as pharmacology at higher doses because bioactive compounds are almost always harmful at high doses [16].

Utami and Yusi [17] indicated that the toxicity test results were expressed as lethal concentration 50 (LC50), which is the optimal concentration of extract that can kill 50% of the *Artemia salina* population. Consequently, a lower LC50 value suggests a more significant hazardous effect. The benefits of this BSLT test are that it is simple, quick, straightforward, repeatable, and inexpensive [18]. Moreover, *Artemia salina* tests are susceptible to harmful chemicals [19].

To evaluate the efficacy of conventional treatments [20], a scientific investigation, such as in the fields of pharmacology, toxicology, the identification, and isolation of active chemical components present in plants is required. Therefore, this study intends to establish the anticancer efficacy of clove flower extract (*Syzygium aromaticum*) by determining grid-score values using molecular docking and LC50 values using the brine shrimp lethality test (BSLT) method.

## 2. Materials and Methods

### 2.1. Study Design of the BSLT Method

This study was an experimental investigation with a post-test-only control group design to determine the toxicity of clove flower extract to *Artemia salina* utilizing the brine shrimp lethality test (BSLT) and molecular docking. The research design had a completely randomized design. This research has also adhered to the guidelines of the Faculty of Veterinary Medicine's ethics commission of Airlangga University.

Ten samples were taken for every concentration of *Artemia salina* larvae. This study prepared five strengths of clove flower extract in six experimental groups. Five repetitions of each concentration and control were conducted. The required sample size is, therefore, 300 larvae.

This research was conducted between February and March 2022 in the Laboratory of the Veterinary Basic Medicine Division, Faculty of Veterinary Medicine, Airlangga University, and Unit Layanan Pengujian (ULP), Faculty of Pharmacy, Airlangga University, to produce clove extract. The ethical Committee of the Faculty of Veterinary Medicine at Airlangga University approved all the procedures for laboratory animals.

### 2.2. Clove Flower Extraction

The clove flower was obtained from a local farm in the Naringgul District, South Cianjur Regency, West Java Province, Indonesia. Whole and dried clove flowers weighed as much as 900 grams. Then, it was processed with a set of distillation tools. First, the distilled extraction liquid was collected, and to separate the essential oil from water, dichloromethane was added to the separating funnel in a ratio of 1 : 3. After accommodating the generated volatile oil, Na2SO4 anhydrous was applied to eliminate the remaining water [21]. Treatment solution was prepared from 20 mg of essential oil dissolved in 2 ml of ethanol, then pipetted into vials as much as 25 *µ*l, 125 *µ*l, 250 *µ*l, 375 *µ*l, and 500 *µ*l, then left for 24 hours to evaporate solvent [22].

### 2.3. Hatching *Artemia salina*

The larvae of *Artemia salina* (Supreme plus® Goldenwest, origin from Great Salt Lake USA) were obtained from the Gunung Sari Ornamental Fish Market in Surabaya, East Java. The hatching of larvae occurs in an aquarium. The environmental conditions for hatching *Artemia salina* larvae are pH 8–9; water salinity between 5 and 70 ppt; and temperatures between 26 and 31 degrees Celsius. At the same time, aeration is regulated by utilizing an aerator.

The aquarium was equipped with a bulkhead whose bottom edge was perforated, allowing hatched eggs to escape through the hole before being refilled with seawater. *Artemia salina* eggs are put into one compartment, which is subsequently sealed. In another area, a 10-watt lamp attracts newly hatched shrimp. 48-hour-old larvae will serve as test subjects [17].

### 2.4. Toxicity Test Using the BSLT Method

One ml of seawater was added to the treatment solution and homogenized with a vortex. We put 10 larvae of *Artemia salina* into the vial and add seawater to a final volume of 5 ml so that the final results of the test solution are obtained with concentrations of 50 ppm, 250 ppm, 500 ppm, 750 ppm, and 1000 ppm. The control group was only given 5 ml of seawater without the clove extract. The toxicity test was repeated five times. Then, observations were made for one day on the death of *Artemia salina*. The mortality results of *Artemia salina* at each concentration were compared with controls. Observations can be made after 24 hours of treatment. The standard criteria for assessing the mortality of *Artemia salina* larvae is that the larvae do not show movement for 10 seconds of observation.

The sample toxicity test was determined by evaluating the value of LC50, which can kill *Artemia salina* up to 50%. Statistical calculations were performed using probit analysis with Statistical Program and Service Solutions (SPSS) for Windows version 25. From the percent mortality, we monitor for the probit number or value of each group of test animals through the table, determine the dose log for each group, and then make a graph with a straightline equation of the relationship between the probit value and the concentration log, *y* = ax + *b*, where *y* = probit number and *x* = concentration log, a line is drawn from probit 5 (=50% mortality) to the *X* axis, and the concentration log is obtained. Concentration logs are antiloged to get LC50, or LC50 values can also be calculated from the straight-line equation by entering the value 5 (probit of 50% of experimental animal deaths) as *y* so that *x* is produced as the log concentration value. LC50 is calculated and obtained from the antilog of the *x* value [23].

### 2.5. Design of Molecular Docking

Molecular docking is conducted using the Dock 6.8 software package. The employed structures are listed in Table 1. The structural and molecular surface preparations were carried out using Chimera 1.16's Dock Prep and Write DMS functions (1). Using the SPHGEN tool, spherical structures were formed on the surface of molecules. Since the precise location of the enzyme's active site is known, the SPHERE SELECTOR tool was used to choose spheres within a radius of 8.0 from the ligand. The SHOWBOX application then generates a simulation box with the correct dimensions for the selected spheres plus a margin of 8.0 in all directions. Using the GRID software, a 0.25 resolution grid was created. Van der Waals interaction was modeled using the Lennard-Jones 12–6 potential, while electrostatic interaction was modeled using the Coulomb potential with a value of = 4r (2). Redocking is used to validate the docking parameters, where the docking approach is considered legitimate if the symmetry-corrected root means the square deviation of the heavy atom (HA RMSDh) is less than 2.0. Using the MMFF approach in the Avogadro software (3), the structures of eugenol and caryophyllene were optimized, and then the AM1-BCC electrostatic charge was applied using the ANTECHAMBER program (4). Visualization of the docked positions utilizing Maestro 12.4 Release 2020–2 (Schrodinger, Inc).

## 3. Results and Discussion

### 3.1. BSLT Result

**Figure 1:**
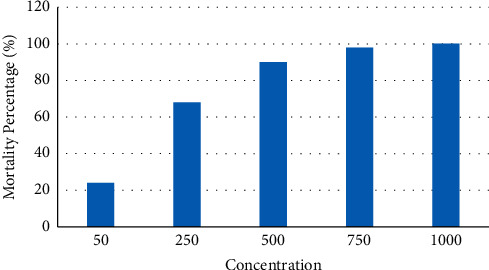
The toxicity test results of the concentration of clove flower extract (*Syzygium aromaticum*) on *Artemia salina* larvae.

In the study of the toxicity test of clove flower extract as a potential anticancer medicine utilizing the BSLT method, it was determined that clove flower extract was poisonous to *Artemia salina* in the BSLT test (see Figure 1). Based on the investigation results, 900 grams of clove flower distillation produced essential oil as 9.75 (w/w) grams, with a yield of 1.083%. This extract proved poisonous to 48-hour-old *Artemia salina*. In T1, where the extract concentration was 50 ppm, the mortality rate was 24% because clove flower extract's toxicity level is still low. The concentration of the pesticide's chemical components within the target species' body significantly impacts the insecticide's toxicity [23]. At T2, with a 250 ppm extract concentration, the mortality rate was 68%. At this dosage, clove extract was poisonous, as it could kill *Artemia salina* by more than 50 percent. At a concentration of 250 ppm, it is possible to determine the LC50 value. In T3, where the extract concentration was 500 ppm, the percentage of death was 90%; in T4, where the extract concentration was 750 ppm, the percentage of mortality was 98%. This result suggests that the clove flower extract is highly toxic to *Artemia salina* at this dose. T5 possessed the most destructive potential, with an extract concentration of 1000 ppm. The proportion of deaths was 100 percent. The rise in extract concentration induces an increase in the number of active components in these substances, which function as insecticides capable of killing numerous species [24].

**Figure 2:**
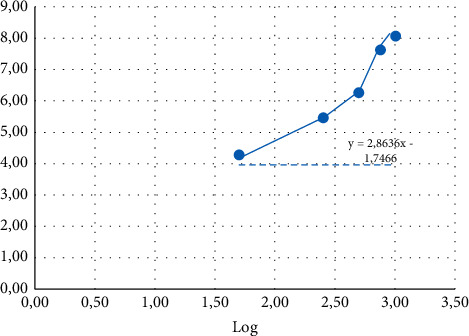
Graph of the correlation of the log concentration of clove flower extract (*Syzygium aromaticum*) with the probit mortality rate of *Artemia salina* larvae.

In the control group, there were no dead larvae since there was no addition of clove flower extract containing poisonous compounds. It demonstrated that the death of *Artemia salina* was caused by the administration of clove flower extract and not by environmental factors. Thus, it can be seen that the number of *Artemia salina* fatalities is proportional to the concentration of clove flower extract. This result is consistent with the findings of Lisdawati et al. [25], Sanjaya et al. [18], and Sapulette et al. [26], who discovered a correlation between the concentration of plant extracts and the number of dead larvae. This research indicates that the number of *Artemia salina*-related fatalities is often proportional to the concentration of clove flower extract. The LC50 of clove flower extract was calculated to be 227.1 g/ml using the probit method. LC50 500–1000 g/ml is mildly toxic, LC 50 100–500 g/ml is highly toxic, and LC 50 0–100 g/ml is extremely poisonous [19]. Based on these factors, the clove extract is classified as moderately hazardous. The level of toxicity provides context for the potential anticancer activities of clove flower extract. The lower the LC 50, the greater the plant's potential as a cancer treatment.

(See Figure 2) provides the equation for the line *y* = ax + *b*, which can be used to confirm the LC50 value. By providing the value of probit 5 (*y* = 5) into the equation y = 2.8636 x – 1.7466 yields the result of x = log 2.356, which is then antiloged to yield the value of 227.1 g/ml. The results of the probit analysis are qualitatively estimated values, so they did not reflect the actual value. Consequently, interval estimates also emerge in the findings of the probit analysis. Based on the investigation, the estimated interval between the values range is 135–293. Figure 2 describes that The *R*2 value is known to be 0.9062, indicating that clove flower extract is 90% effective at killing *Artemia Salina*. The coefficient of determination (*R*2) is a component of the total diversity of the dependent variable *Y*, which may be accounted for or explained by the variety of the independent variable *X*. The value of R2 is between 0 and 1, and it is argued that the correlation improves as R2 approaches 1 [27]. In the toxicity test, the *R*2 value is 1, indicating that the clove flower extract administered is the cause of the mortality of the test larvae.

*Artemia salina* larvae exhibit a high level of sensitivity to testing. Based on their morphology, 48hour-old *Artemia salina* has developed a mouth and digestive system to absorb specific particles. Despite having a digestive system, *Artemia salina* larvae that are 24 hours old or in the second instar phase, cannot interact with their surroundings and cannot absorb extracts or external substances [19].

In order to achieve *Artemia salina* egg hatching and growth, salinity, pH, and seawater temperature must be considered. This investigation utilized seawater with a salinity of 37 ppt. The pH of the used seawater is 8.1. A pH fall below 7 can be fatal. [28] Hatching cysts require a pH of 8–9, which is slightly alkaline. In this investigation, the seawater temperature was 28.3⁰C. *Artemia salina* cannot thrive at temperatures below 6°C or above 35°C in hatcheries. The optimal Artemia growth temperature varies from 26 to 31 degrees Celsius [29]. During the hatching process, a lamp is added to the *Artemia* larvae as a light source to maintain the optimal water temperature and to stimulate the larvae to shed their eggshells. The actively moving larvae swim toward the light source due to their positive phototaxis features that attract light [30].

*Artemia salina's* digestive tract is a nonselective filter, which makes it easier for poisonous chemicals to enter the mouth. Phyto-chemicals from clove flower extract can interact with cell membrane targets and enzymes, resulting in death [31]. It is in agreement with the initial research studies that demonstrate the eugenol component of clove extract acts as a larvicide against *Artemia salina* by damaging cell membranes or interfering with the metabolism of the larvae [32, 33].

The brine shrimp lethality technique (BSLT) is a leading method for evaluating the presence of bioactive compounds in natural products, with results typically related to cytotoxic and anticancer activity [34]. Meyer et al. [35] initially elucidated this method, 48 hours after being soaked in brine, brine shrimp eggs will begin to hatch. Each plant extract is diluted in 20 ml of a mixture of methylene chloride and methanol to form a stock solution with a 10 mg/ml concentration (1 : 1). Then, aliquots of 500, 50, and 5 g/ml are transferred in triplicate from the stock solutions to the vials, and the solvent is allowed to evaporate. After evaporation, 5 ml of brine are added to each vial to generate 1000, 100, and 10 ppm concentrations. Each vial is filled with ten nauplii (30 shrimps per concentration). Lethal concentrations at 50% mortality (LC50) values are determined using the Finney computer program's number of survivors at each concentration.

As a preliminary anticancer screening test, the BSLT method is insufficient for determining the mechanism of action of bioactive substances in plants and is not specific for anticancer activity. However, the BSLT method generates data that can be supported by more specific bioassays after the active compounds tested are toxic to *Artemia salina*, indicating that they are likely potential candidates for anticancer research. Further evaluation to indicate cytotoxicity of *Syzygium aromaticum* can be conducted in vitro by 3-[4,5-dimethylthiazol-2-yl]-2,5 diphenyl tetrazolium bromide (MTT) assay [36], to consider the safety of the product application in the human model cells.

### 3.2. Molecular Docking

Clove essential oil includes many components, with eugenol, eugenol acetate, and caryophyllene constituting the majority. The three chemicals were analyzed in silico by molecular docking with nine proteins essential for cancer cell proliferation [7]. These three compounds can be evaluated by comparing their grid score to the original ligand's grid score (cognate). The outcomes are shown in Table 2. According to these findings, no substance can produce a higher grid score than cognate due to the clove essential oil's bioactive structure, including a small number of functional groups, particularly caryophyllene, which is rich in hydrophobic interactions. Other than the van der Waals interaction, there are few other possible interactions. In addition, caryophyllene produced the lowest grid score on nine target proteins compared to the other two chemicals.

**Figure 3:**
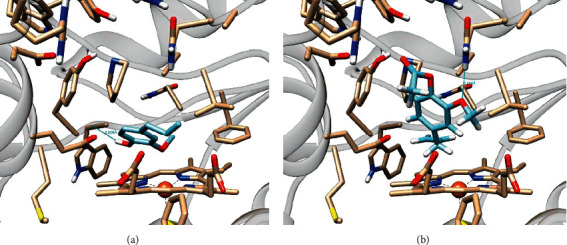
Docking pose eugenol (a) and (b) eugenol acetate on the active site of the NO synthase enzyme.

Eugenol acetate has the closest grid score to AR-C95791 (-42.12 vs. -61.27) in the NO synthase enzyme compared to other ligands and targets. This molecule can maintain hydrogen bond contacts between oxygen in methyl ether and Gln181 residue when examined from the docking stance. The fragment allyl points to heme (Figure 3(a)). Eugenol has a comparable grid score (-41.5). However, his docking position is the opposite. The phenolic group in eugenol points to heme, and the phenolic OH group interacts with the Tyr291 residue's backbone (Figure 3(b)). Although not as effective as its homolog, eugenol can inhibit the production of NO by this enzyme [33]. Eugenol may also be created as a NO synthase inhibitor by including multiple functional groups to enhance the number of contacts through a series of chemical modifications.

## 4. Conclusion

The clove flower extract was determined to be moderately toxic to *Artemia salina* in the BSLT test with an LC50 value of 227.1 g/ml based on probit analysis. Hence, it has the potential to be developed as an anticancer medicine. In addition, eugenol acetate is close to its cognate enzyme nitric oxide synthase based on its proximity to the grid-score value (3E7G). Therefore, if in vitro test results indicate that cancer cell proliferation is inhibited, the most likely mechanism is the suppression of nitric oxide synthase.

## Figures and Tables

**Table 1:** Crystal structures were used.

No	Access code PDB	Description	Resolution	Reference (DOI)
1	2JF9	Estrogen receptor alpha LBD in complex with a tamoxifen-specific peptide antagonist	2.10 Å	10.1074/jbc.M611 424200
2	1HOV	Solution structure of a catalytic domain of MMP-2 complexed with SC-74020	NMR	10.1016/s0167-4838(02)00307–2
3	1UK0	Crystal structure of the catalytic domain of human poly (ADP-ribose) polymerase with a novel inhibitor	3.00 Å	10.1016/s0014-5793(03)01362–0
4	2A4L	Human cyclin-dependent kinase 2 in complex with roscovitine	2.40 Å	10.1111/j.1432–1033.1997.0518a.x
5	2R0U	Crystal structure of Chek1 in complex with inhibitor 54	1.90 Å	10.1016/j.bmcl.2007.09.007
6	2 × 7F	Crystal structure of the kinase domain of human Traf2- and nck- interacting kinase with Wee1Chk1 inhibitor	2.80 Å	—
7	3E7G	Structure of human INOSOX with inhibitor AR-C95791	2.20 Å	10.1038/nchembio. 115
8	3RUK	Human cytochrome P450 CYP17A1 in complex with abiraterone	2.60 Å	10.1038/nature107 43
9	4LXD	Bcl_2-navitoclax analog (without thiophenyl) complex	1.90 Å	10.1038/nm.3048
10	6GUE	CDK2/CyclinA in complex with AZD5438	1.99 Å	10.1016/j.chembio l.2018.10.015

**Table 2:** Grid-score results and hydrogen bond interactions.

Protein	Ligand	Grid-score	H-bond number	Residue (H-Bond length, Å)
Estrogen receptor-*α*	Eugenol	−29.758898	2	Lys145 (2.455); Ile82 (2.637)
	Eugenol acetate	−34.403477	0	
	Caryophyllene	−18.799114	0	
	Tamoxifen	−**66.970085**	1	Glu49 (1.954); Arg90 (2.046)
MMP-2	Eugenol	−41.468246	0	
	Eugenol acetate	−43.793243	1	Ser151 (2.388)
	Caryophyllene	−34.205738	0	
	SC-74020	−**109.474319**	4	Leu83 (2.017); Ala84 (2.441); His120 (3.054); Glu121 (2.421)
PARP	Eugenol	−31.103073	1	Ser243 (2.205)
	Eugenol acetate	−36.013237	1	Ser203 (2.585)
	Caryophyllene	−32.597618	0	
	FR257517	−**63.837357**	0	
CDK2	Eugenol	−36.910698	2	Asp74 (1.845); Lys77 (1.815)
	Eugenol acetate	−41.676758	1	Lys33 (2.136)
	Caryophyllene	−40.535130	0	
	Roscovitine	−**71.813713**	2	Leu71 (2.572; 1.992)
Chk1 kinase	Eugenol	−38.836273	3	Glu44 (1.504); Asn48 (2.658); Asp137 (1.801)
	Eugenol acetate	−39.099670	1	Asp137 (2.447)
	Caryophyllene	−35.456528	0	
	Cpd. 54	−**84.449051**	5	Glu74 (1.844); Cys76 (1.793); Glu80 (1.634); Glu123 (2.297); Asp137 (1.966)
NO synthase	Eugenol	−41.503204	1	Tyr291 (2.208)
	Eugenol acetate	−42.120934	1	Gln181 (2.406)
	Caryophyllene	−35.420425	0	
	AR-C95791	−**61.271812**	2	Tyr265 (2.129); Glu295 (1.937)
Human	Eugenol	−29.011292	0	
Cytochrome	Eugenol acetate	−32.352654	0	
P450	Caryophyllene	−31.890057	0	
CYP17A1	Abiraterone	−**61.819771**	1	Asn172 (1.864)
BCL-2	Eugenol	−35.300983	0	
	Eugenol acetate	−36.267906	0	
	Caryophyllene	−28.939451	0	
	ABT-199	−**79.199051**	1	Asn82 (2.186)
Cyclin A	Eugenol	−29.981400	2	Lys34 (2.082); Asp141 (2.623)
	Eugenol acetate	−31.090176	0	
	Caryophyllene	−25.151398	0	
	AZD5438	−**60.700954**	4	Lys34 (2.228); Leu79 (2.197); Leu79 (2.333); Asp82 (1.946)

## Data Availability

The data used to support the findings of this study are available from the corresponding author upon request.
